# Is there Any Correlation Between Nasal Height and Facial–Nasal Horizontal Anthropometrics? An Anthropometric Analysis Using Three-Dimensional CT

**DOI:** 10.1007/s00266-026-05729-y

**Published:** 2026-03-06

**Authors:** Alperen Can Kokten, Umut Tuncel, Ayhan Sonmez, Mehmet Kadir Atdagi

**Affiliations:** Department of Plastic Reconstructive and Aesthetic Surgery, Medical Faculty, Samsun University, 55100 Samsun, Turkey

**Keywords:** Facial anthropometrics, Nasal anthropometrics, Nasal height, Rhinoplasty

## Abstract

**Background:**

The relationship between horizontal nasal and facial anthropometric measurements and nasal height remains unaddressed, and normative data specific to the Turkish population are currently limited in the existing literature.

**Materials and Methods:**

A review of nasal bone anthropometrics was conducted using three-dimensional computed tomography (3D-CT) images from 249 randomly selected adult Turkish patients who applied for rhinoplasty in 2025. In this cross-sectional study, high-resolution CT scans were reconstructed into three-dimensional views employing standardized imaging parameters. The mean bizygomatic distance (K), the widest length of nasal aperture (L), the narrowest length of the nasal aperture (M), the width at nasion level (N), and the nasal height (O), also key anthropometric ratios (K/O, L/O, M/O, N/O), were calculated and compared across sex and nasal bony shape groups (S vs V-shaped). Receiver operating characteristic (ROC) analysis was conducted to establish discriminatory thresholds, with particular emphasis on nasal height.

**Results:**

Of the cases, 141 were male, and 108 were female. A total of 178 patients had an S-shaped configuration, while 71 patients had a V-shaped configuration. Male patients had significantly higher nasal bone ratios than females (p < 0.05). No statistically significant differences were observed between S- and V-shaped morphologies. All horizontal measurements were positively correlated with nasal height. ROC analysis identified nasal height as a moderate discriminator, with a threshold of 1.65 cm. The main finding was that the K/O ratio was the most potent determinant for planning nasal height. Additionally, a formula was developed to estimate the ideal dorsal projection, assessing nasal height and its proportion relative to overall facial width.

**Conclusions:**

The study’s results indicate that as horizontal dimensions increase, nasal height also increases, a phenomenon that is more pronounced in men. Additionally, the most critical determinants were the bizygomatic width and the widest nasal aperture length, which can be used to estimate the desired nasal height for surgical planning in rhinoplasty.

**Level of Evidence III:**

This journal requires that authors assign a level of evidence to each article. For a full description of these Evidence-Based Medicine ratings, please refer to the Table of Contents or the online Instructions to Authors  www.springer.com/00266.

## Introduction

It has been demonstrated that the nasal skeleton plays a central role in both facial aesthetics and function. Its morphology plays a pivotal role in determining nasal profile, dorsal contour, and airway support, making accurate assessment essential for successful rhinoplasty outcomes. Nasal bone morphology has been described in the literature as S- and V-shaped nasal bones, and this finding has been widely accepted and used by many authors since it was first introduced by Lazovic et al [1, 2]. Variations in nasal bone shape and size are influenced by sex, ethnicity, and environmental factors, and documenting these differences has long been of interest to both surgeons and anthropologists.

Historically, nasal bone measurements have been derived from surface anthropometry or two-dimensional radiographs. While such approaches provided valid descriptive data, they were limited by projection errors and a lack of three-dimensional accuracy. With the advent of high-resolution computed tomography (CT), it has become possible to obtain precise morphometric data and reconstruct the nasal framework in three dimensions. Several studies have utilized CT to analyze nasal bones in various populations, including those from East Asia, the Middle East, and Europe [1–4]. To date, the study with the most comprehensive series utilizing 3D-CT was conducted by the senior authors in 2025 [5]. In this study, a positive correlation was found between dorsal profile angle and nasal bone configuration. Additionally, the study has proven the three-dimensional computed tomography method to be very useful for this type of anthropometric study. In addition, the results obtained from this study shed light on whether there is any correlation between horizontal facial and nasal anthropometric measures and nasal height. Thus, the authors conducted the current study, which also includes ratio-based analyses in addition to basic measurements, enabling a more standardized comparison of nasal morphology by minimizing variability related to overall size. These ratios may also have greater clinical significance in making decisions about procedures and predicting postoperative stability. Using receiver operating characteristic (ROC) analysis on nasal bone morphology further increases its translational value by providing cut-off points that can be incorporated into surgical decision-making.

Recent developments in the field of rhinoplasty have led to renewed interest in nasal bone anthropometry. There is a growing body of the literature that recognizes the importance of determining the nasal bone morphology. However, most of these studies have focused on specific angles of the nasal bone. Still, other aspects have also been overlooked, and there has been no detailed investigation of the probable relationship between horizontal facial and nasal dimensions and nasal height. The present study aims to provide a comprehensive evaluation of nasal bone morphology in a Turkish series using three-dimensional computed tomography (3D-CT) imaging. It was conducted to explore the potential relationship between horizontal nasal and facial dimensions and nasal height. By calculating morphometric ratios, comparing sex and shape-based differences, and exploring predictive thresholds through ROC analysis, we sought to establish population-specific normative data and highlight their clinical implications in rhinoplasty.

## Materials and Methods

This cross-sectional study included adult patients who underwent high-resolution computed tomography for nasal indications. The study was approved by our institution’s ethics committee (GOKAEK 2025/15/6). All procedures were conducted in accordance with the ethical standards of the institutional and national research committees, as well as the 1964 Declaration of Helsinki and its subsequent amendments or comparable ethical standards. Patients with severe nasal trauma, congenital nasal deformities, or a history of secondary rhinoplasty were excluded from the study. Poor-quality imaging constituted an additional exclusion criterion.

CT imaging was performed using a 64-slice Philips Ingenuity scanner (Philips Healthcare, The Netherlands). Standardized parameters included 120 kVp, a rotation time of 1.2 seconds, a table feed of 0.5 mm/sec, and a slice thickness of 1.5 mm. Images were processed through the FONET PACS system and reconstructed into three-dimensional views on an AWS server (version 3.2 ext 3.0). This protocol ensured high-resolution visualization of the nasal skeleton and reproducible morphometric analysis.

### Definitions and Measurements

**Fig. 1:**
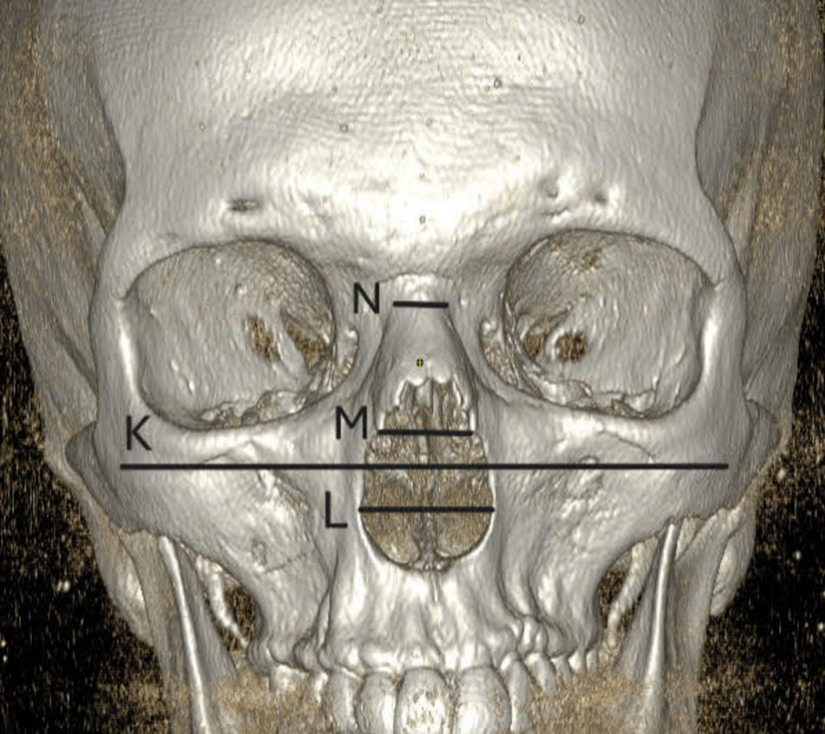
In the S-shaped nasal bony vaults, an anterior 3D CT reconstruction illustrating measurement points K (bizygomatic width), L (the widest length of the nasal aperture), M (the narrowest length of the nasal aperture), and N (width at nasion level)

**Fig. 2:**
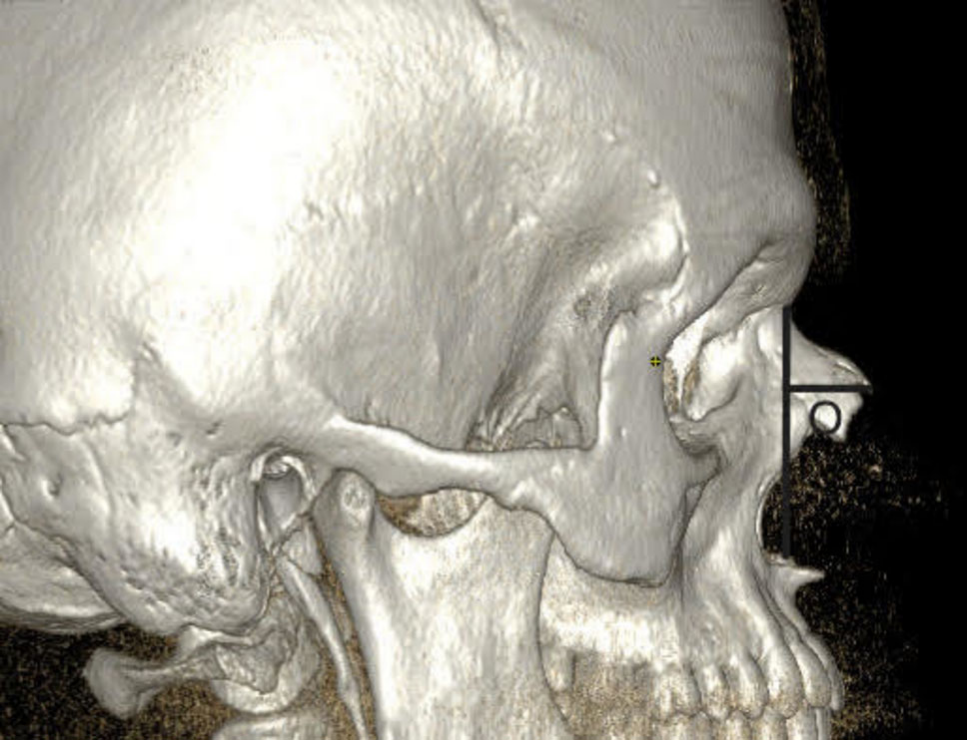
Three-dimensional CT reconstruction from the lateral view showing measurement of nasal bone height (O) in the S-shaped nasal bony vaults

**Fig. 3:**
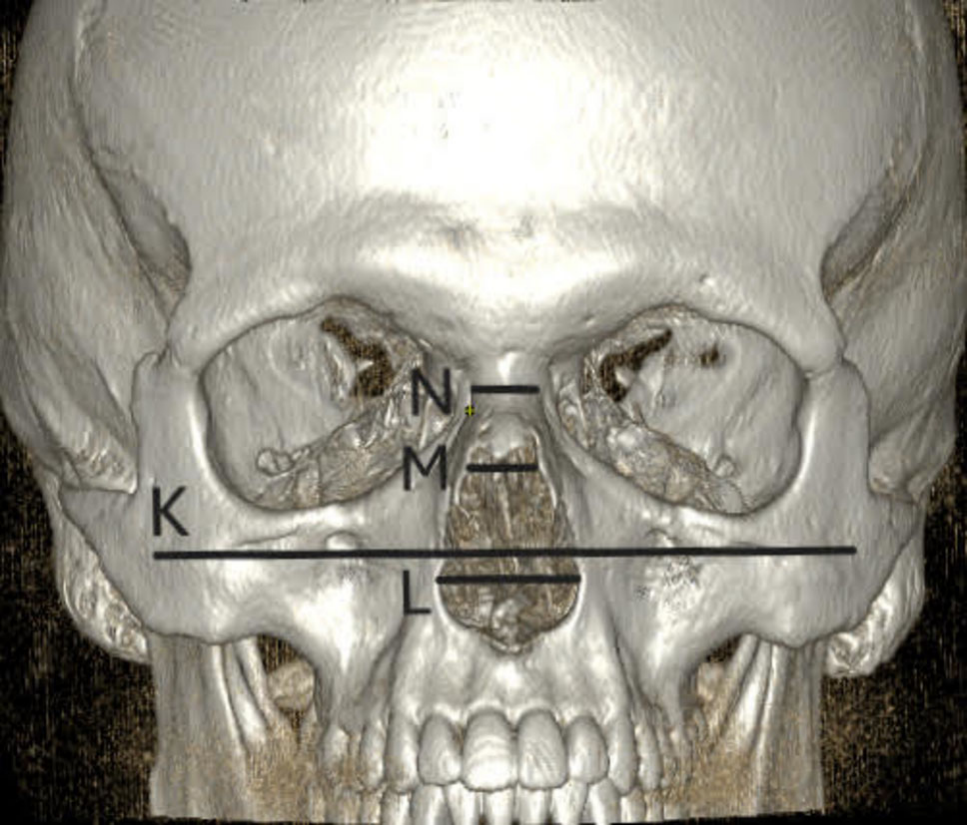
In the V-shaped nasal bony vaults, an anterior 3D CT reconstruction illustrating measurement points K (bizygomatic width), L (the widest length of the nasal aperture), M (the narrowest length of the nasal aperture), and N (width at nasion level)

**Fig. 4:**
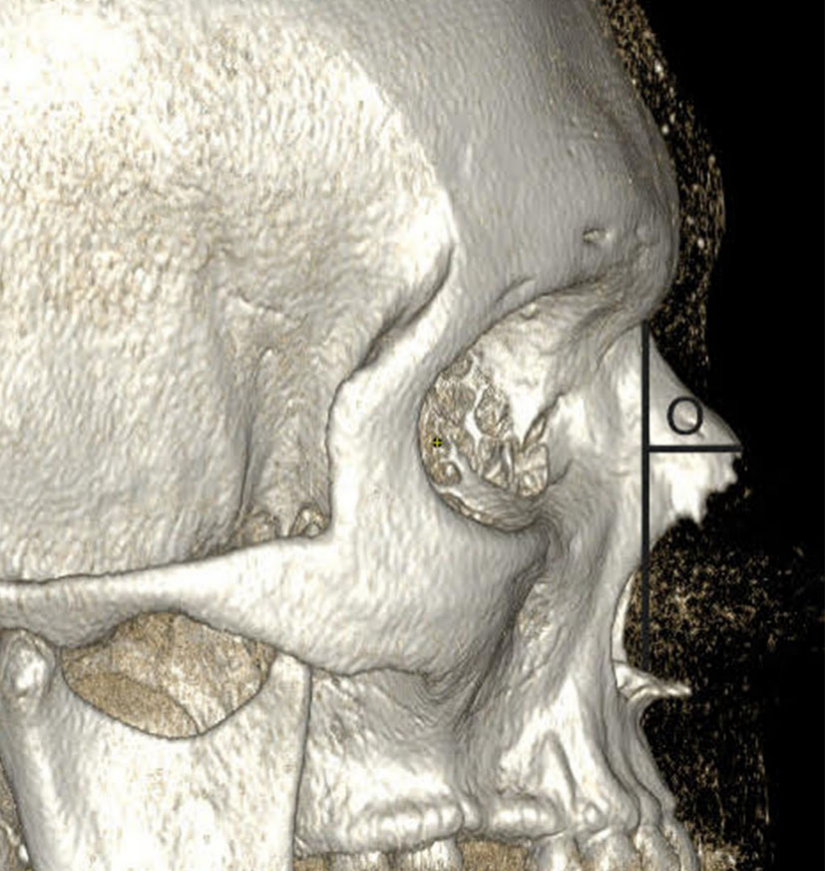
Three-dimensional CT reconstruction from the lateral view showing measurement of nasal bone height (O) in the V-shaped nasal bony vaults

Figure 1 shows an anterior 3D-CT reconstruction illustrating measurement points K (bizygomatic width), L (the widest length of the nasal aperture), M (the narrowest length of the nasal aperture), and N (width at nasion level). Figure 2 shows a three-dimensional CT reconstruction from the lateral view, showing measurement of nasal bone height (O) in S-shaped nasal bones. Figure 3 shows an anterior 3D-CT reconstruction illustrating measurement points K (bizygomatic width), L (the widest length of the nasal aperture), M (the narrowest length of the nasal aperture), and N (width at nasion level). Figure 4 shows a three-dimensional CT reconstruction from the lateral view, showing measurement of nasal bone height (O) in V-shaped nasal bones.

Four key ratios were calculated to standardize nasal bone morphology:

K/O ratio: bizygomatic width to nasal height,

L/O ratio: the widest length of the nasal aperture to nasal height,

M/O ratio: the narrowest length of the nasal aperture to nasal height,

N/O ratio: the width at the nasion level to nasal height.

## Statistical Analysis

Descriptive statistics were expressed as means ± standard deviation or medians with interquartile ranges, as appropriate. Between-group comparisons were performed using independent t- tests or Mann–Whitney U tests. A Spearman correlation test was used to analyze the relationship between horizontal anthropometrics and nasal height. Receiver operating characteristic (ROC) analysis was applied to assess the discriminatory capacity of morphometric parameters, with area under the curve (AUC) values reported. Statistical significance was set at *p*< 0.05. A multiple linear regression analysis was conducted to determine the effects of the variables K, L, M, and N, which were considered potential predictors of the dependent variable O. Sample size adequacy was evaluated for correlation and regression aims. With n=249 and *α*=0.05 (two-tailed), the study provides high power to detect small-to-moderate correlations (e.g., *r*≈0.20) and ensures stable estimation for a multiple linear regression model with four predictors. Nasal bone height (O) was defined as a single standardized midline measurement on 3D-CT (not side-specific).

## Results

**Fig. 5:**
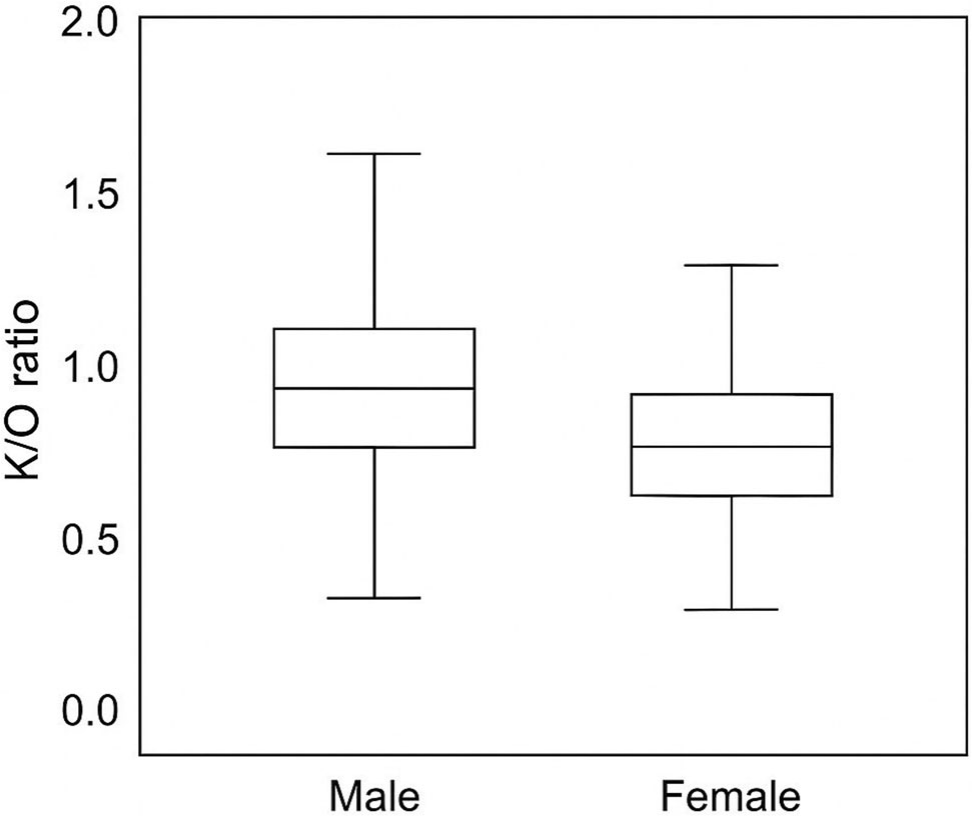
Distribution of K/O ratio by gender (boxplot)

**Fig. 6:**
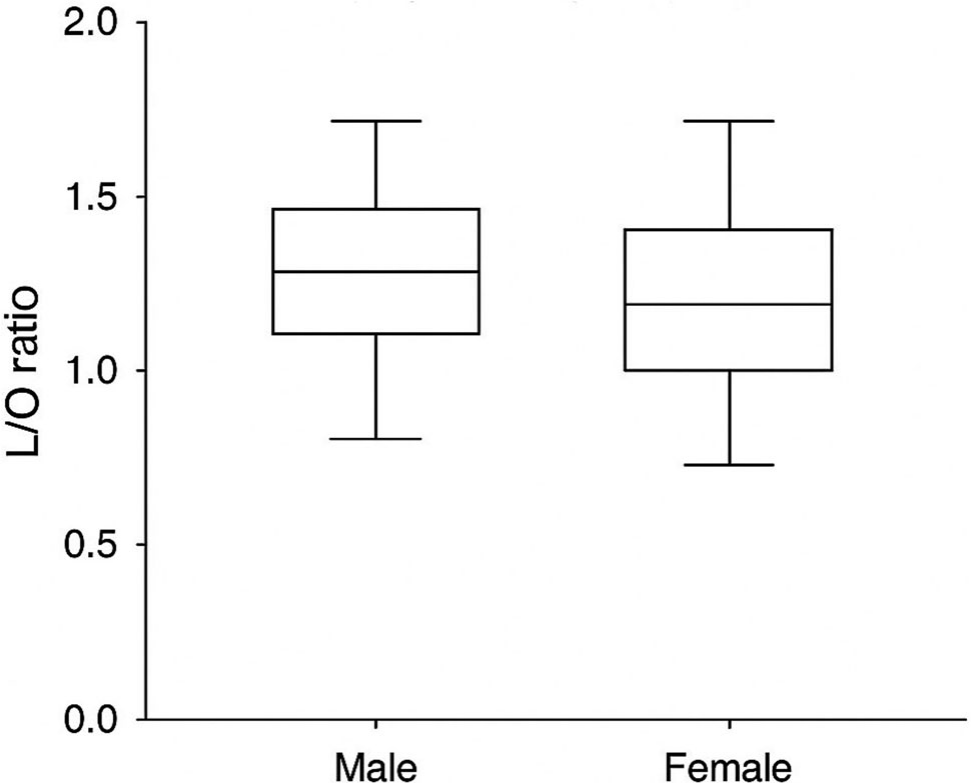
Distribution of L/O ratio by gender (boxplot)

**Fig. 7:**
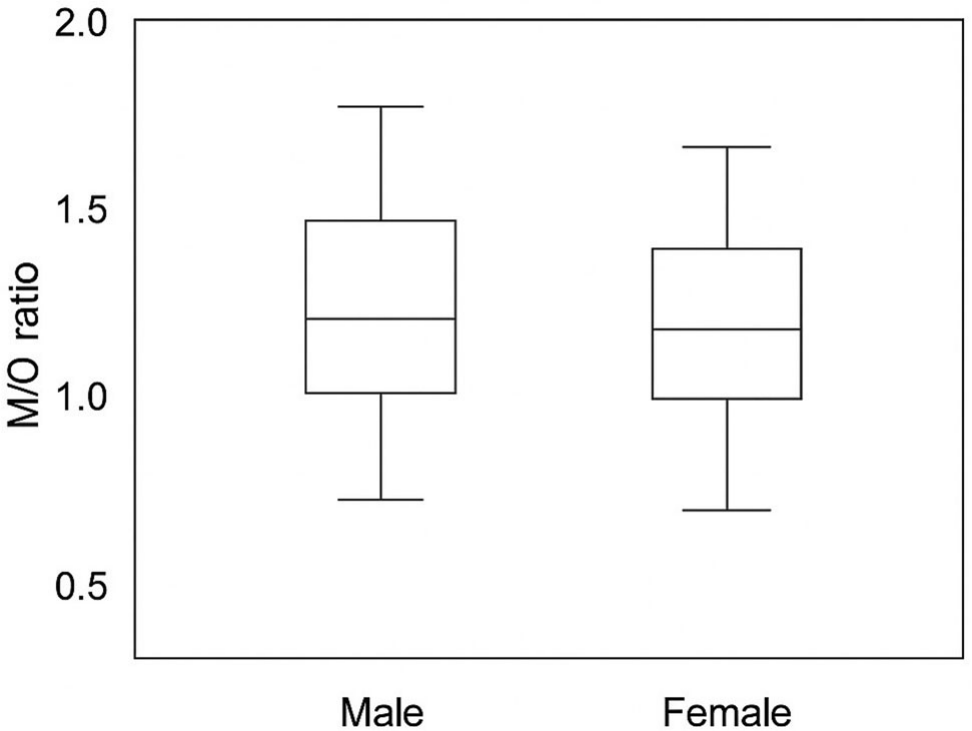
Distribution of M/O ratio by gender (boxplot)

**Fig. 8:**
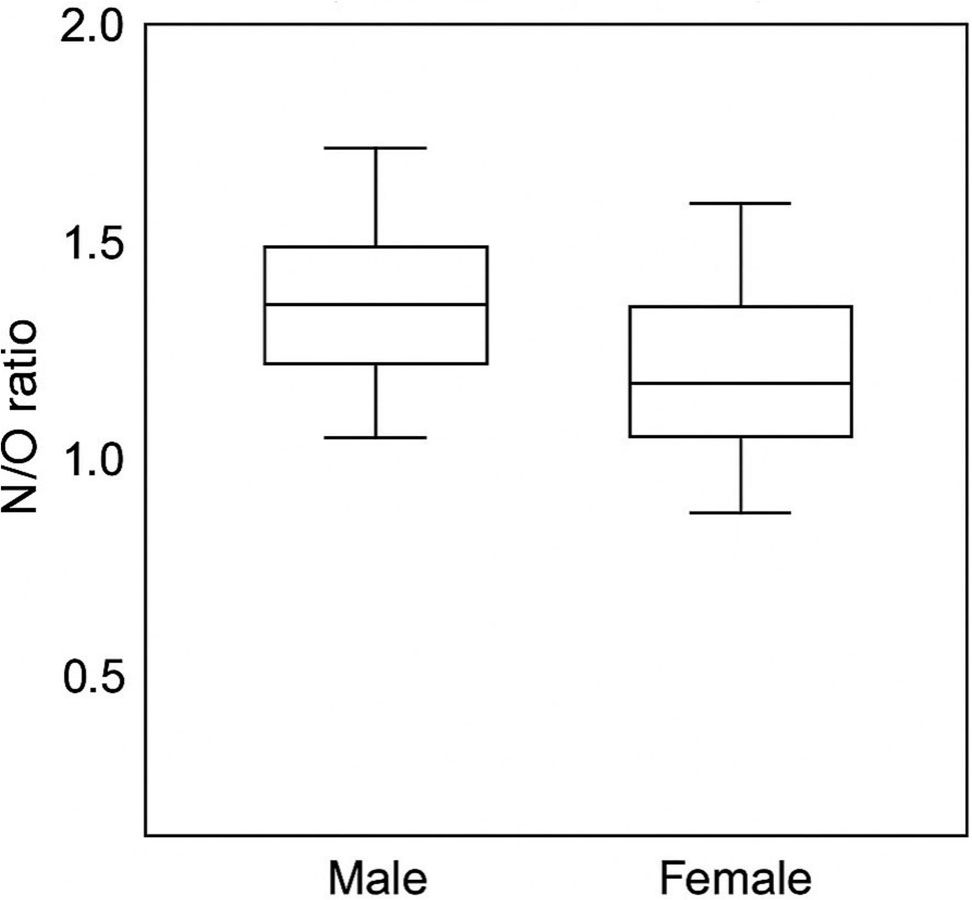
Distribution of N/O ratio by gender (boxplot)

**Fig. 9:**
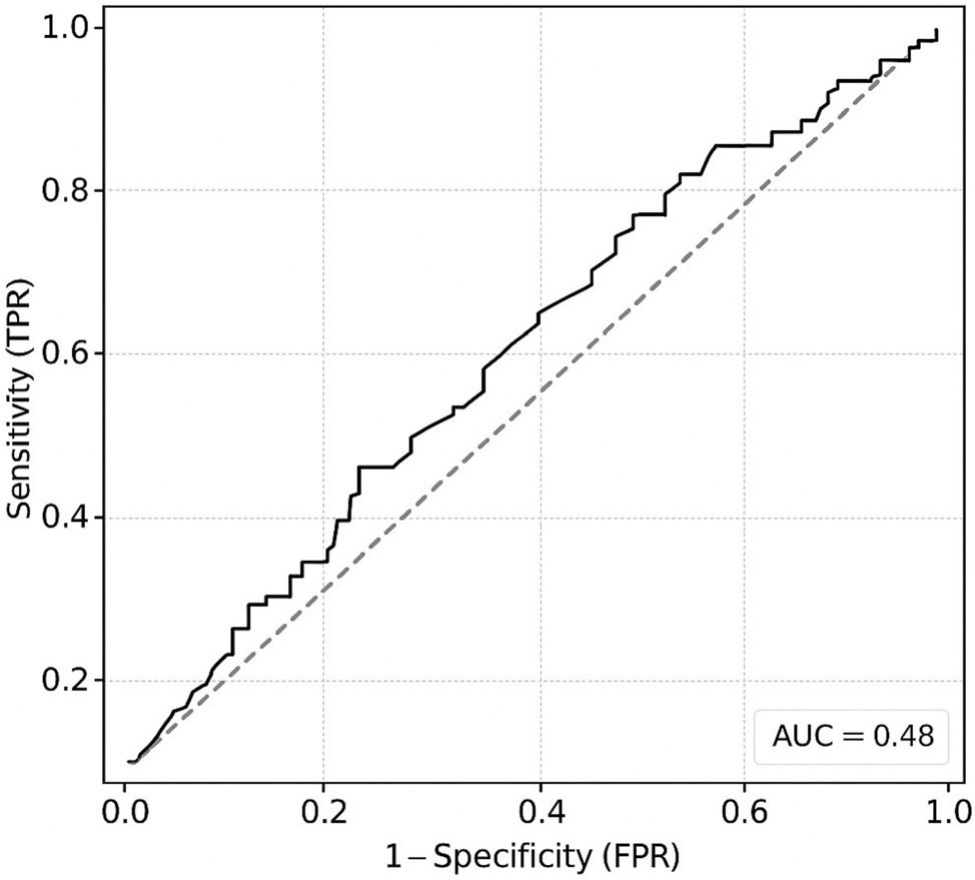
ROC analysis (Python)

A total of 249 patients were included in the study (male, 141 [56.6%]; female, 108 [43.4%]). Regarding nasal morphology, 178 patients (71.5%) had an S-shaped configuration, and 71 patients (28.5%) had a V-shaped configuration. The mean bizygomatic distance (K) was 11.62 ± 0.93 cm, the widest length of the nasal aperture (L) 2.45 ± 0.27 cm, the narrowest length of the nasal aperture to nasal height (M) 1.54 ± 0.24 cm, the width at nasion level (N) 1.06 ± 0.65 cm, and the nasal height (O) 1.64 ± 0.28 cm. When comparisons were made according to gender, all ratios (K/O, L/O, M/O, N/O) were significantly higher in males than in females (*p* < 0.05, Table 1, Figs. 5, 6, 7, 8, 9). In contrast, when it was made according to the nasal shapes, no significant differences were observed in the ratios between the S-shape and V-shape groups (*p* > 0.05, Table 1). Only the L/O ratio showed a trend toward higher values in the V-shape group, but this did not reach statistical significance.

**Table 1:** Descriptive statistics and group comparisons (SPSS results)

Variable	Male (Mean ± SD)	Female (Mean ± SD)	*p* (M vs F)	*p* (S vs V)
K/O ratio	0.145 ± 0.022	0.136 ± 0.019	0.002	Ns
L/O ratio	0.698 ± 0.128	0.645 ± 0.116	<0.001	Ns
M/O ratio	1.122 ± 0.226	1.038 ± 0.195	0.002	Ns
N/O ratio	1.700 ± 0.350	1.520 ± 0.296	<0.001	Ns

K/O ratio: bizygomatic width to nasal height, L/O ratio: the widest length of the nasal aperture to nasal height, M/O ratio: the narrowest length of the nasal aperture to nasal height, N/O ratio: the width at the nasion level to nasal height

Spearman correlation analysis demonstrated that K, L, M, and N measurements were positively correlated with O (nasal height) (r = 0.14–0.45, all *p* < 0.05, Table 2). This finding indicates that greater horizontal distances are associated with greater nasal height.

**Table 2:** Correlation analyses (SPSS Spearman results)

Variable	O (nasal height) r (p)
K (Bizygomatic)	0.453 (*p*<0.001)
L (Aperture wide)	0.142 (*p*=0.013)
M (Aperture narrow)	0.215 (*p*<0.001)
N (Nasion narrow)	0.296 (*p*<0.001)

Receiver operating characteristic (ROC) analysis was performed to determine potential cut-off thresholds for differentiating nasal shape types (Table 3). Among the examined parameters, O (nasal height) demonstrated poor discriminative ability, with an AUC of 0.52 and an optimal cut-off value of 1.65 cm (sensitivity, 0.46; specificity, 0.65). This indicates that nasal height alone cannot reliably distinguish between S- and V-shaped nasal bones. Other morphometric parameters similarly exhibited AUC values close to 0.5, suggesting limited predictive potential. For gender differentiation, no clinically significant thresholds were identified (AUC ≈ 0.55–0.65). A multiple linear regression analysis was conducted to determine the effects of the variables K, L, M, and N, which were considered potential predictors of the dependent variable O. According to the analysis results, the overall model was found to be statistically significant (*F* = 21.3, *p* <.001). The coefficient of determination (*R*^2^ = 0.259) indicates that approximately 25.9% of the total variance in the dependent variable O is explained by the independent variables included in the model (Table 4).

**Table 3:** ROC analysis (Python)

Parameter	AUC	Cut-off (cm)	Sensitivity	Specificity	Interpretation
O (Nasal height)	0.52	1.65	0.46	0.65	Poor discriminative ability

**Table 4:** A multiple linear regression analysis

Predictor	*B*	SE	*t*	*Β*	95% CI		*p*	VIF	Tolerance
					Lower	Upper			
Intercept	−0.0360	0.1971	−0.183				<.001		
K	0.1786	0.0227	7.875	0.5903	0.4427	0.7380	<.001	1,85	0.541
L	−0.1931	0.0758	−2.548	−0.1862	−0.3302	−0.0423	0.011	1,76	0.569
M	0.0271	0.0781	0.347	0.0228	−0.1067	0.1523	0.729	1,42	0.703
N	0.0297	0.0238	1.248	0.0690	−0.0399	0.1780	0.213	1,01	0.993
Dependent Variable: O					R^2^=0,259	F=21,3			

K (Bizygomatic)

L (Aperture wide)

M (Aperture narrow)

N (Nasion narrow)

To assess the presence of multicollinearity among predictor variables, the variance inflation factor (VIF) and tolerance values were examined. The results indicated that VIF values ranged from 1.01 to 1.85, while tolerance values ranged from 0.541 to 0.993.

Since all variables met the accepted thresholds (VIF < 2 and tolerance > 0.10), it can be concluded that no multicollinearity problem exists in the model, and the predictors contribute independently to explaining the variance in O.

### Clinical Interpretation of the Predictive Formula for Nasal Height

The multiple linear regression model demonstrated that nasal height (O) can be reliably predicted from transverse facial parameters, particularly the bizygomatic width (K) and the nasal aperture width (L). The derived equation:

$$O\_{\mathrm{predicted}} = - 0.0360 + \left( {0.1786 \times K} \right) - \left( {0.1931 \times L} \right) + \left( {0.0271 \times M} \right) + \left( {0.0297 \times N} \right)$$

*Variable K*: Exhibits a statistically significant positive effect on the dependent variable (B = 0.1786, *t* = 7.875, *p* <.001). This indicates that, holding other variables constant, a one-unit increase in *K* leads to an approximate increase of 0.1786 units in *O*.

*Variable L*: It has a significant negative effect (*B* = −0.1931, *t* = −2.548, *p* = 0.011)—accordingly, a one-unit increase in *L* results in an average decrease of 0.1931 units in *O*.

*Variable M*: Although it has a positive coefficient (*B* = 0.0271), this effect is not statistically significant (*p* = 0.729). Therefore, *M* does not appear to have a meaningful impact on *O* within the model.

*Variable N*: Shows a positive direction of effect (*B* = 0.0297), but it is not statistically significant (*p* = 0.213).

## Discussion

Anthropometric evaluation of the nasal region has a long history, dating back to early surface-based measurements in physical anthropology. Classical approaches relied on calipers and cephalometric radiographs, which offered only limited two-dimensional representations of a complex three-dimensional structure. Over the past two decades, advances in CT and digital imaging have shifted the field toward high-resolution morphometry. Our study reflects this progression, building upon earlier descriptive and cephalometric work by introducing ratio-based 3D-CT analysis supported by ROC methodology. Most prior studies have highlighted ethnic and sex-based variability in nasal morphology. Alharethy et al. [2] demonstrated sexual dimorphism in a Middle Eastern population, while Lee et al. [3] and Kim et al. [5] analyzed East Asian cohorts using CT. Lazovic et al. [2] highlighted the anatomical importance of nasal bone dimensions in aesthetic surgery, and Ludwigs et al. [6] provided normative data for Eurasians. Our results align with these studies by confirming the male predominance in nasal bone dimensions in Turkish populations; however, we extend the literature by introducing ratio-based parameters and ROC-derived thresholds.

Numerous studies in the literature describe the morphology of the nasal bone based on its angles. [5–17]. In the most comprehensive series on nasal bone anthropometrics recently published, the authors investigated how nasal bone angles vary between two different nasal shapes using 3D-CT [1]. In this study, statistically significant associations between nasal bone morphology and its angles have been found. However, the main limitation of this study was that only the nasal bone was documented, and the angles of the nasal bone alone were studied. In the current study, the morphology of the nasal bone was examined based on facial, nasal horizontal measurements, and nasal height, in conjunction with the surrounding bone structure.

Numerous studies have shown that nasal morphology might differ significantly due to ethnic factors. This is the first report on facial–nasal ratio based from a nationally representative cohort of patients. Turkish series remain understated in the literature, despite the country’s unique position bridging Europe and Asia. By generating normative data for a Turkish sample, our study addresses this gap. These findings enhance the surgical relevance of anthropometric data for local patients and serve as reference values for comparative international research. The higher ratios observed in males reflect not only an overall larger bone size but also greater lateral support. Although no statistically significant differences were observed between S- and V-shaped morphologies, trends in L/O ratios among V-shaped noses suggest narrower bony apertures. Clinically, this may translate into differences in dorsal aesthetics, hump reduction, and internal valve function. Even subtle variations in shape could influence patient-specific outcomes, underscoring the importance of documenting and further investigating these distinctions in larger case series. Anthropometric variation carries both functional and aesthetic implications. Narrower apertures and disproportionate bone ratios may predispose patients to nasal obstruction, particularly after osteotomy or dorsal modification. Incorporating ratio-based analysis into preoperative planning could help reduce the risk of iatrogenic airway compromise and improve functional outcomes. Future studies correlating morphometric ratios with objective airflow measurements could provide further insight into these functional relationships.

Among the studies that utilize dimensional data of the nasal bone and the surrounding facial bones [18–23], the presented study has some significant and distinctive features compared to the similar ones. First, this study benefits from methodological objectivity. By using a software program, we achieved high-resolution, reproducible measurements. Standardized parameters reduced inter-individual variability, and 3D reconstruction minimized projection errors inherent in 2D methods. This technical foundation represents a substantial improvement over traditional anthropometry, enhancing both research reliability and clinical translation. Second, the introduction of ROC analysis represents a novel contribution. Previous studies typically reported mean values without defining cut-off thresholds. By applying ROC methodology, we identified nasal height (O) as a potential predictor of nasal morphology, with a threshold of 1.65 cm. Although AUC values were moderate, this step advances nasal bone anthropometry from descriptive reporting to actionable clinical criteria, thereby bridging the gap between research and practice. Multiple linear regression was chosen to quantify the independent contributions of K, L, M, and N to nasal height (O) and to derive a predictive equation. Model assumptions (linearity, residual normality, homoscedasticity, influential outliers, and independence) were assessed using residual diagnostics; multicollinearity was evaluated using VIF and tolerance. ROC analysis was used to evaluate discriminatory performance and to propose clinically interpretable cut-off values for binary outcomes; AUC with 95% CI was reported. And third, translating our findings into clinical practice is straightforward. The study employed regression analysis to develop a practical formula for estimating the ideal nasal height (O) based on facial and nasal horizontal dimensions. This approach provides a fast and reproducible intraoperative reference for assessing the proportionality of dorsal projection relative to overall facial width. It was observed that individuals with wider facial proportions tend to have greater nasal height. Accordingly, males generally demonstrate higher nasal projection. No significant difference was found between S- and V-shaped facial ratios. Based on our study of 249 patients, we developed a clinical formula. This formula was established through correlation and regression analyses. According to these analyses, as shown in Table 4, the most determinative variables for nasal height were the bizygomatic distance (K) and the widest nasal aperture length (L). Therefore, bizygomatic distance, the widest length of the nasal aperture, and nasal height values were incorporated into this formula. When applied to 249 cases, the formula indicated that patients with values below 0.20 cm would likely need augmentation, whereas those with values above 0.20 cm would require dorsal reduction rhinoplasty. However, it is evident that, in clinical decision-making, not only the bony framework but also the skin and soft- tissue thicknesses play an essential role in determining the final surgical approach. Preoperative CT provides not only anatomical visualization but also quantitative ratios (K/O, L/O, M/O, N/O). Applying threshold-based evaluations of nasal height allows surgeons to stratify patients into risk groups and adjust osteotomy plans accordingly. This framework may also enhance preoperative discussions with patients by offering objective, quantifiable data to support shared decision-making. The clinical relevance of this formula lies in its ability to estimate nasal height in relation to facial width. The model is derived from horizontal facial anthropometric measurements and reflects the underlying bony framework, independent of soft tissue effects. In rhinoplasty practice, decisions regarding dorsal reduction or dorsal augmentation are often based on visual assessment and surgical experience. This formula provides an approximate, patient-specific numerical reference for the amount of nasal height that may be decreased or increased. By entering facial width measurements, surgeons can obtain an expected nasal height that is proportionate to the face. This information may help define reasonable upper and lower limits for bony dorsal modification during preoperative planning. The formula does not prescribe an aesthetic result or replace clinical judgment. Instead, it offers objective, skeletal-level guidance that may support a more controlled, proportionate adjustment of nasal height. Final aesthetic decisions remain dependent on surgical technique, intraoperative findings, and soft tissue response.

## Limitations

Despite these strengths, several limitations must be noted. In this study, because measurements were performed by a single trained evaluator using predefined anatomical references and a standard measurement protocol, a second evaluator and a repeat-measurement round could not be created, so ICC could not be calculated. Instead, we detailed the measurement steps to increase methodological rigor and emphasized standardization to minimize possible measurement error (same software, same imaging parameters, same magnification/calibration approach, blinding, etc.). The study has no age-related findings. ROC performance, while promising, requires validation in larger populations. Radiation exposure remains a limitation; however, modern CT protocols and cone-beam CT (CBCT) may help mitigate this concern. Additionally, our analysis focused exclusively on bony structures; integrating soft tissue parameters could provide a more comprehensive understanding of nasal morphology.

## Conclusion

The study set out to investigate the relationship between facial–nasal horizontal measurements and nasal height. Our results indicate that greater horizontal distances are associated with greater vertical nasal bone height. This study provides novel insights into nasal bone morphology within a Turkish cohort, utilizing three-dimensional computed tomography and quantitative anthropometric analysis. Our findings confirm significant sex-related differences in nasal bone ratios, whereas S- and V-shaped morphologies showed no statistically significant differences. Notably, nasal height (O), bizygomatic (K), and the widest aperture distances (L) demonstrated moderate discriminatory capability in regression analysis, thereby supporting their potential utility as clinically relevant parameters in rhinoplasty planning.
